# Gastrointestinal Physiology Before and After Duodenal Switch with Comparisons to Unoperated Lean Controls: Novel Use of the SmartPill Wireless Motility Capsule

**DOI:** 10.1007/s11695-021-05452-4

**Published:** 2021-05-08

**Authors:** Khalid Elias, Per M. Hellström, Dominic-Luc Webb, Magnus Sundbom

**Affiliations:** 1 Department of Surgical Sciences, Uppsala University, SE-751 85 Uppsala, Sweden; 2 Department of Medical Sciences, Uppsala University, SE-751 85 Uppsala, Sweden

**Keywords:** Gastric emptying, Transit time, Motility index, Hunger contractions, GSRS

## Abstract

**Purpose:**

Bariatric surgery alters gastrointestinal anatomy. In this exploratory study, the SmartPill® wireless motility capsule (WMC) was used to study changes in gastrointestinal physiology following biliopancreatic diversion with duodenal switch (BPD/DS).

**Material and Methods:**

Twenty-eight BPD/DS patients (35 ± 11 years, 50% females, body mass index [BMI] 56 ± 5) were to be examined preoperatively and postoperatively. In addition to transit time, appetite control and gastrointestinal symptoms were studied by patient-scored questionnaires (visual analogue scale and Gastrointestinal Symptom Rating Scale (GSRS)). Data was compared to 41 lean unoperated controls.

**Results:**

About 1.8 years postoperatively, 18 patients (BMI 35.8 ± 8.3) returned for a second WMC test. As expected, small bowel transit time was reduced, from 3.9 ± 1.6 h to 2.8 ± 2.0, *p* = 0.02, and at both these time points, it was shorter than in lean controls (5.4 ± 1.9 h, *p* = 0.001). Postoperatively, a trend towards reduced colon and whole gut transit times was seen in BPD/DS-patients, thus approaching those of lean controls. Surprisingly, BPD/DS patients scored higher satiety than controls preoperatively as well as increased hunger and desire to eat postoperatively. Compared to lean, BPD/DS patients reported a higher total GSRS score at both time points (1.2 ± 0.2 vs 1.7 ± 0.6 and 2.3 ± 0.5, *p* < 0.001). Postoperatively, the scores for diarrhea and indigestion increased.

**Conclusions:**

The novel use of the SmartPill system in BPD/DS patients gave the expected readouts. Although small bowel transit time was further shortened after BPD/DS, whole gut transit time did not differ from controls. Typical gastrointestinal symptoms were reported postoperatively.

**Graphical abstract:**

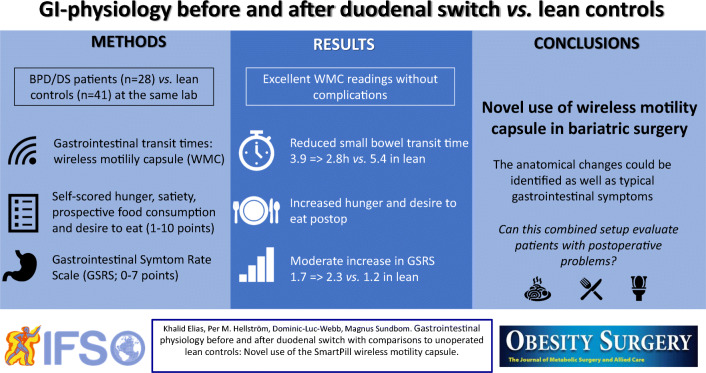

**Supplementary Information:**

The online version contains supplementary material available at 10.1007/s11695-021-05452-4.

## Introduction

In an effort to reduce excess weight, bariatric surgery changes gastrointestinal anatomy. Physiological effects on bowel function and observed gastrointestinal symptoms differ depending on procedure. In general, Roux-en-Y gastric bypass reduces acid-related conditions, while reflux can be aggravated by sleeve gastrectomy [1]. Biliopancreatic diversion with duodenal switch (BPD/DS), often used in patients with severe obesity, defined as body mass index (BMI) ≥ 50 kg/m^2^ [2], has a marked impact on bowel function due to small bowel shortening. Although improving physical quality of life, BPD/DS is known to increase the daily number of bowel movements and the risk for troublesome flatus [3, 4]. At present, this is considered an inevitable consequence of the shortened small bowel, resulting in only a 1–1.5 m common channel for fat absorption.

In order to better understand and optimize bariatric procedures, it is important to study all types of physiological changes after surgery. Gastrointestinal transit times can be studied by scintigraphy and radio-opaque markers [5]. Although scintigraphy requires minimal radiation, whole gut studies take considerable time to complete [6]. Radio-opaque markers are therefore more widely used. However, this modality cannot be used to measure the transit time of a physiological meal [7]. A wireless motility capsule (WMC) system is capable of measuring gastric emptying time as well as small bowel and colon transit times [8]. Use of the SmartPill® WMC in bariatric patients has not been reported to date.

The primary aim of this study was to investigate physiological changes in the gastrointestinal tract following BPD/DS. WMC transit time data was compiled along with patient-scored questionnaires. A secondary aim was to compare preoperative and postoperative WMC measurements to lean controls.

## Methods

### Study Cohort

Twenty-eight subjects (38.4 ± 11.3 years, 14 females) with a BMI of 56.5 ± 5.1 were continuously recruited at our outpatient clinic for WMC testing and evaluation of patient-rated gastrointestinal symptoms before and at least 1 year after BPD/DS. The results were compared to 41 lean subjects (28.4 ± 12.8 years, 21 females, BMI 22.6 ± 2.1) who had been investigated previously at our lab [9].

Minimally invasive BDP/DS was performed through five trocars with a 5-mm liver retractor. After dividing the duodenal bulb 2–3 cm distal to the pylorus, a gastric sleeve was constructed over a 35-Fr boogie, starting the linear stapling 4–5 cm proximal to the pylorus. Using the double omega-loop technique, a 100-cm common limb of distal ileum and a 150-cm alimentary limb were constructed and anastomosed side-to-end to the proximal part of the duodenal bulb by two running sutures. A side-to-side entero-enterostomy between the distal part of the jejunum, i.e., the end of the biliopancreatic limb, and the most proximal part of the common limb restored continuity [10]. Finally, the small bowel segment between the two anastomoses was divided, creating a Roux-en-Y construction. Both mesenteric defects were closed by one row of metallic clips^.^

### WMC Recordings

The single-use, 26 × 13 mm cylindrical WMC encapsulates sensors, a radio transmitter, and a battery, providing power for at least 5 days [11]. The system records temperature (25 to 49 °C), pH (0.05 to 9.0 units), and pressure (0 to 300 mmHg), which are continuously transmitted to a receiver unit. Subjects arrived in the fasting state in the morning and received a standardized meal containing 260 kcal (75% carbohydrates with 3% fiber, 21% protein, and 3% fat) [9]. Thereafter, they swallowed the WMC together with 150 ml tap water. Subjects were instructed to be ambulatory and to start eating ad libitum 6 h after the capsule ingestion. They were also instructed to carry the wireless data receiver in a sling around their neck during waking hours and to keep it close to the body until passage of the WMC. [12].

Gastrointestinal transit times were defined by characteristic changes in temperature and intraluminal pH. Gastric emptying time (GET) was defined as the time from ingestion of WMC (i.e., transition to ~37 °C) to pyloric passage (an abrupt rise in pH, exceeding 3 units from gastric baseline, to pH > 4). Small bowel transit time (SBTT) was defined as time from pyloric passage to ileocecal junction (pH rise > 1.5 units), while the remaining time to exit (abrupt pH drop of approximately one unit and temperature drop to room temperature, or signal loss) was defined as colon transit time (CTT). The sum of these three parts equaled whole gut transit time (WGTT) [13].

Intraluminal pressures in the stomach (corpus and antrum) and small bowel were studied. Motility Index (MI) values, calculated as MI = Ln (sum of amplitudes × number of contractions + 1), were analyzed for separate 30-min intervals just before and after WMC passage through pylorus [14] as well as ileo-cecal valve. Hunger contractions, a part of the migrating motor complex (MMC), were defined as 3–5 contractions exceeding an amplitude of >50 mmHg, within 5 min prior to gastric emptying of the WMC. Median and lowest gastric pH was determined in the BPD/DS patients as acid-related conditions, e.g., reflux and stomal ulcers [15], are associated with bariatric surgery.

### Appetite Control

Four 100-mm visual analogue scales (VAS) measuring hunger, satiety, prospective food consumption, and desire to eat were used to assess subjective appetite sensations [16]. Appetite control was documented at 10 min before, immediately after (zero), 60, 120, and 180 min after WMC ingestion.

### Gastrointestinal Symptom Rating Scale

The Gastrointestinal Symptom Rating Scale (GSRS) is a validated disease-specific instrument depicting reflux, abdominal pain, indigestion, diarrhea, and constipation. The GSRS has a 7-point graded scale where 1 represents absence of symptoms and 7 represents very troublesome symptoms [17, 18]. GSRS was administered to BPD/DS patients before and after surgery and lean controls.

### Statistical Analysis

Data is presented as mean and standard deviation, unless specified otherwise. Normally distributed data were compared by Students *t*-test, Kruskal-Wallis test for VAS, while paired sample Wilcoxon signed ranks test for the remaining variables. Correlations between BMI and the results of the three investigations were studied by Spearman’s rank correlation. Percent excess BMI loss (%EBMIL) was defined as ([baseline BMI − BMI after surgery]/[baseline BMI − 25]) × 100. Percentage total body weight loss (%TWL) was calculated by kilograms lost divided by the starting weight × 100. MotilityGI version 3.0 software was used to generate graphs for visual assessment and summary reports of computed transit times in hours and minutes. SPSS version 26 (IBM, Armonk, NY) was used for statistics.

## Results

### Study Participation and Weight Loss

As stated in Table 1, 18 of the 28 (64%) BPD/DS patients returned for a second WMC, when having lost 34 ± 13% of their former weight, corresponding to a %EBMIL of 67.2 ± 23.4%. The WMC examinations were performed without any complications. BPD/DS subjects were somewhat older than controls, 35.0 ± 11.3 vs 28.4 ± 12.8 years and had higher preoperative and postoperative BMI (56.5 ± 5.1 and 35.8 ± 8.3 vs. 22.6 ± 2.1, *p* < .05 for all).

**Table 1:** Characteristics for the BPD/DS patients and lean controls. Data are in mean ± SD

	BPD/DS		*p*	Lean controls	*p*	*p*
	Preop (*n* = 28)	Postop (*n* = 18)		(*n* = 41)	Lean vs preop	Lean vs postop
Patient demographics						
Age (y)	35.0 ± 11.3	36.8 ± 11.1	<.005	28.4 ± 12.8	<.005	.019
Gender m/f, (% female)	14/14 (50.0)	8/10 (44.4)	.713	21/20 (51.2)	.558	.632
Obesity-related diseases, *n* (%)						
Diabetes	10/28 (35.7%)	0/18 (0%)	.031	-		
Hypertension	12/28 (42.9%)	3/18 (16.7%)	.250	-		
Dyslipidemia	3/28 (10.7%)	0/18 (0%)	.999	-		
Osteoarthritis	7/28 (25%)	4/18 (22.2%)	.999			
BMI (kg/m^2^)	56.5 ± 5.1	35.8 ± 8.3	<.005	22.6 ± 2.1	<.005	<.005
%EBMIL	-	67.2 ± 23.4		-		
%TWL	-	34.3 ± 13.4		-		
Time to second visit (years)	-	1.8 ± 0.7		-		

Obesity-related diseases are defined by pharmacological treatment. %EBMIL and %TWL denote percent excess BMI loss and percent total weight loss, respectively

### Transit times

**Fig. 1:**
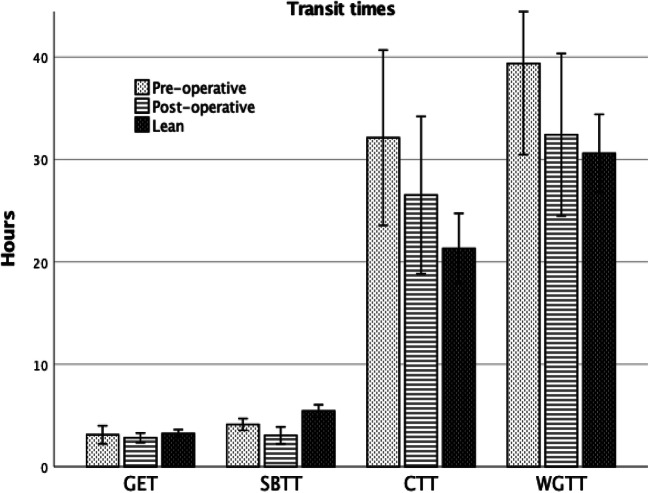
Gastrointestinal transit times; gastric emptying time (GET), small bowel transit time (SBTT), colon transit time (CTT), and whole gut transit time (WGTT) in preoperative and postoperative BPD/DS patients compared to lean controls. Values in mean, error bars: Standard error of the mean

GET did not differ after surgery, nor in patients with or without diabetes at baseline. Rapid gastric emptying (<1.5h) was observed in three patients before surgery and in one patient after surgery. Three patients had slow GET (>4h) before surgery, but none after surgery. SBTT became shorter after surgery, 2.8 ± 2.0 vs. 3.9 ± 1.6 h. Compared to controls, BPD/DS patients had a faster SBTT, both preoperatively and postoperatively (*p* < 0.05 for all). The remaining transit times, CTT and WGTT, did not differ, although a trend was seen towards normalization of both after BPD/DS (*p* = 0.071 and 0.051, respectively) (Fig. 1; Table 2).

**Table 2:** SmartPill results concerning transit times, intraluminal pressure, motility index, hunger contractions, and gastric pH for the BPD/DS group and lean controls. Data are in mean ± SD

	BPD/DS		*p*	Lean controls	*p*	*p*
	Preop (*n* = 28)	Postop (*n* = 18)		(*n* = 41)	Lean vs preop	Lean vs postop
Gastrointestinal transit times (h)						
GET	3.2 ± 2.7	2.9 ± .8	.632	3.2 ± 2.7	.959	.154
SBTT	3.9 ± 1.6	2.8 ± 2.0	.022	5.4 ± 1.9	.001	<.001
CTT	31.5 ± 25.0	25.4 ± 16.3	.112	21.3 ± 10.8	.051	.264
WGTT	38.6 ± 26.0	31.1 ± 16.8	.071	30.6 ± 12.1	.136	.897
Intraluminal pressure (mmHg)						
Gastric corpus	3.8 ± 4.3	3.0 ± 1.0	.492	3.5 ±1.2	.039	.266
Gastric antrum	4.3 ± 5.0	4.4 ± 2.1	.492	4.4 ± 2.3	.125	.609
Small bowel	4.5 ± 1.6	3.4 ± 1.2	.066	4.0 ± 1.1	.141	.142
Motility index						
Proximal to the pylorus	81.6 ± 61.6	90.1 ± 74.9	.663	85.2 ± 68.7	.875	.818
Distal to the pylorus	142.9 ± 132.6	98.2 ± 130.6	.500	123.9 ± 109.3	.419	.098
Proximal to the ileo-cecal value	262.5 ± 223.5	136.5 ±143.9	.011	254.0 ± 231.4	.627	.012
Distal to the ileo-cecal value	189.3 ± 213.2	110.3 ± 87.1	.260	180.4 ± 208.5	.683	.559
Hunger contractions						
Presence, (%)	12/26, (46.2)	6/13, (46.2)	.999	19/40, (47.5)	.915	.933
Gastric pH						
Lowest pH	0.2 ± 4.0	1.5 ± 1.6	.161	.6 ± .3	.127	.018
Median pH	2.6 ± 1.6	2.9 ± 2.2	.632	1.2 ± 1.1	<.001	<.001

Motility index was studied during 30-min intervals just before and after the capsule had passed the pylorus and ileo-cecal value, respectively

*GET* gastric emptying time, *SBTT* small bowel transit time, *CTT* colon transit time, *WGTT* whole gut transit time

In unoperated individuals, a negative correlation between BMI and SBTT was seen; i.e., increasing BMI reduced transit time (*r* = −0.37, *p* = 0.002). No other correlations were found (BMI vs GSRS domains, %EBMI loss vs GSRS domains post-operative, BMI vs GET, BMI vs CTT).

### Intraluminal Pressure, Motility Index, and pH

A trend towards lower small bowel pressure (*p* = .066) and a reduced ileal MI (*p* < .05) was seen postoperatively. No differences were seen in the remaining variables (gastric pressures, antral MI, hunger contractions, or pH) in BPD/DS patients. In relation to the lean, median gastric pH was higher in the obese both pre operative and postoperative and also slightly higher gastric pressure in obese pre-operative (*p* < .05) as compared to controls.

### Appetite Control

**Fig. 2:**
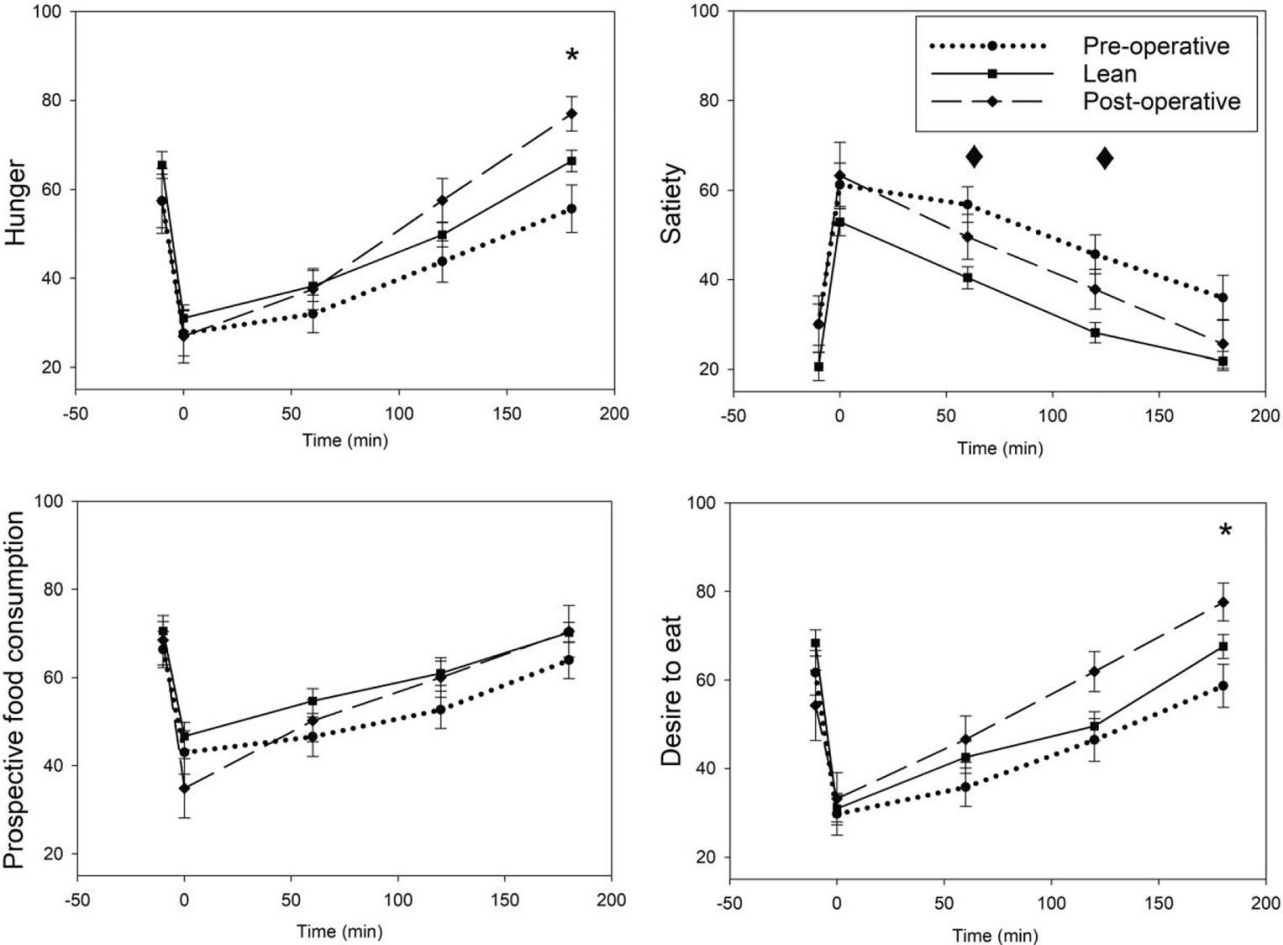
Visual analogue scales (VAS 0–100) demonstrating appetite control (hunger, satiety, prospective food consumption, and desire to eat) in BPD/DS patients preoperatively (*n* = 28) and postoperatively (*n* = 18) as well as lean controls (*n* = 41). Values in mean, error bars: Standard error of the mean. Asterisk and diamond denote *p* < 0.05 between preoperative and postoperative BPD/DS patients and lean vs preoperative BPD/DS patients, respectively

Postoperatively, BPD/DS patients scored higher hunger ratings at 180 min (*p* = 0.020) as well as an increased desire to eat (*p* = 0.035). Compared to lean subjects, BPD/DS patients reported higher satiety scores at 60–180 min preoperatively (Fig. 2; Supplementary Table 1).

### Gastrointestinal Symptom Rate Scale

BPD/DS patients reported increased problems within the Diarrhea and Indigestion domains relative preoperative scores (*p* < 0.05). Before itemizing the GSRS results into the five symptom clusters, patients reported increased abdominal pain, troublesome flatus, loose stools, and early satiety (*p* < 0.05 for all). Compared to controls, the BPD/DS patients reported higher scores throughout all domains (reflux not examined) both before and after surgery (*p* < 0.05) (Table 3).

**Table 3:** Results of Gastrointestinal Symptom Rating Scale (GSRS) in preoperative and postoperative BPD/DS patients compared to our lean controls. Values in mean (SD)

	BPD/DS preop	BPD/DS postop	*p*-value pre vs post	Lean controls	*p*-value pre vs lean	*p*-value post vs lean
Abdominal pain	1.7 ± 0.7	1.9 ± 0.6	.548	1.2 ± 0.5	.013	< .001
Reflux	1.3 ± 0.7	1.5 ± 0.9	.483	-	-	-
Diarrhea	2.0 ± 1.0	3.0 ± 1.3	.007	1.2 ± 0.5	< .001	< .001
Indigestion	1.7 ± 0.6	2.7 ± 0.8	.025	1.2 ± 0.4	< .001	< .001
Constipation	1.7 ± 1.1	2.1 ± 1.3	.444	1.0 ± 0.1	< .001	< .001
Total mean score	1.7 ± 0.6	2.3 ± 0.5	.108	1.2 ± 0.2	< .001	< .001

## Discussion

In this exploratory study, an anticipated rapid small bowel transit after BPD/DS was detected. Concerning the whole gut transit time, operated patients came closer to lean controls, thus demonstrating a high degree of gastrointestinal adaptation. Postoperatively, the typical gastrointestinal symptoms in BPD/DS such as loose stools and diarrhea were noted, however, at a rather modest level.

The SmartPill® WMC system has been proven to correlate with scintigraphy assessment of whole gut transit [6], as well as with the use of radiopaque markers [19]. The SmartPill WMC is approved by the FDA, and the American and European Societies for Neurogastroenterology and Motility have outlined the clinical indications for the use of WMC. Previous studies using different methods have shown contradictory results regarding gastric emptying in patients with obesity. Some have reported faster GET in subjects with obesity and overweight [20], while others, including the present study, have not seen any difference [21]. In operated obese patients, Braghetto et al. showed faster gastric emptying (by scintigraphy) post sleeve gastrectomy, when stapling around a 32 French tube and starting 2 cm from the pylorus [22], while Bernstine et al. using the same method did not see any difference when preserving more of the antrum [23]. The latter is in line with our results and the current surgical technique. A preserved pyloric function in BPD/DS is also believed to protect against pancreatico-biliary reflux into the sleeve.

The faster SBTT seen preoperatively compared to the lean controls could be explained by a larger and more frequent food intake [24] and high-fat diet [25] in the obese group. This is also supported by Gallagher et al, who reported significantly enhanced small bowel contractility in obese patients when comparing smooth muscle cells obtained at routine operations [26]. The release of the gastrointestinal peptide hormone ghrelin is associated with contractile activity of the gastrointestinal tract. In particular, hunger contractions coordinated by ghrelin are believed to clear food residues from the stomach in preparation for the next meal [14]. However, despite an expected reduction in ghrelin due to the resection of the fundus, no drop in hunger contractions was found postoperatively or relative lean controls. This could be interpreted as a sign of preserved gastric motility in the sleeve. Finally, the tendency for the lower intraluminal pressure in the small bowel and patient ratings of increased hunger 2–3 h after WMC ingestion post-surgery might be due to shortened SBTT. The postoperative trend for reduced CTT essentially resulted in normalization of WGTT, making BPD/DS-operated patients similar to lean controls. Moreover, the reduced ileal MI after surgery could be interpreted as an adaptation mechanism, allowing nutrients and electrolytes to be absorbed in the short common limb.

Postoperative satiety for BPD/DS patients came close to lean controls probably a result of the metabolic change. It is, however, difficult to explain higher satiety between unoperated obese patients and lean controls as previous studies show that obese subjects tolerate a larger gastric capacity [27, 28]. A study by Connolly et al. studied the differences in brain responses between lean and obese women and showed a reduced hedonic response in the obese [29], which may explain some of the above-mentioned observations. The pattern of decreasing satiety, and increase hunger, prospective food consumption, and desire to eat after surgery is probably due to the fast propagation of food through the bowel.

Despite higher GSRS scores, compared to lean and preoperative values, the BPD/DS-operated reported a low level of discomfort for all symptoms (≤3), resulting in a total mean GSRS score of only 2.3 on the seven-graded Likert scale. This is in line with Boerlage et al. reporting a total mean score of 2.3 in 168 postoperative gastric bypass patients [30]. However, as expected, our BPD/DS patients scored more discomfort concerning diarrhea postoperatively. Interestingly, no signs of increased gastroesophageal reflux or other acid-related symptoms were seen in the present study. This implies that the patient selection was optimal (no preoperative hiatal hernia or gastroesophageal reflux disease) and that kinking of the sleeve or migration into the thorax was rare.

### Strengths and Limitations

The longitudinal comparisons and inclusion of lean controls studied under identical circumstances at our lab are among the strengths of this study. The difference in age between BPD/DS patients and controls may be of concern. However, a study by Madsen and Graff demonstrated that normal aging (28 to 37 years) does not affect gastric and small bowel motility [31]. Although only 18 BPD/DS patients returned for their postoperative examination, we could still demonstrate both the anticipated changes in gastrointestinal physiology and verify typical gastrointestinal symptoms in BPD/DS, the latter by using the validated GSRS questionnaire. Moreover, we believe that our results are generalizable as the present improvement in obesity-related diseases and weight loss after BPD/DS were comparable to previous studies [32]. The WMC was not able to categorize certain contractile patterns in the small and large bowel since it is a single floating sensor, itself propagating along the bowel [8].

In conclusion, the novel use of the SmartPill WMC system in BPD/DS patients was tolerable for the patients and gave expected readouts in the severely obese. WMC seems suitable for evaluation of gastrointestinal motility disorders, both preoperatively and postoperatively, in this group of patients. The faster small bowel transit time, found in subjects with severe obesity already at baseline compared to lean controls, was increased after BPD/DS. Concerning the whole gut transit time, operated patients came closer to unoperated lean controls, while the reduced ileal motility index demonstrates a high degree of gastrointestinal adaptation. The increased problem with diarrhea in BPD/DS patients after surgery could be verified in the questionnaires.

## Supplementary Information

ESM 1 (DOCX 15 kb).
