# Supplementary material for: Gastrointestinal Physiology Before and After Duodenal Switch with Comparisons to Unoperated Lean Controls: Novel Use of the SmartPill Wireless Motility Capsule
Source: Obes Surg. 2021 May 8;31(8):3483–9. doi: 10.1007/s11695-021-05452-4 (PMC8270844; doi:10.1007/s11695-021-05452-4)
Supplement: Supplementary file 1 — (DOCX 15 kb). [file 11695_2021_5452_MOESM1_ESM.docx]

Supplementary Table 1. Appetite control by visual analogue scale rating (0-100) for hunger, satiety, prospective food consumption and desire to eat. Time in relation to SmartPill ingestion. Values are mean ±SD. P-values in bold font are significant after Bonferroni correction.

|  | Time | -10 min | Zero | 60 min | 120 min | 180 min |
| --- | --- | --- | --- | --- | --- | --- |
| ***Hunger*** | ***p-value between groups*** | .430 | .169 | .639 | .225 | **.022** |
|  | Pre-operative | 57.4 ± 31.8 | 27.6 ± 26.4 | 31.9 ± 22.4 | 43.8 ± 24.3 | 55.7 ± 27.3 |
|  | Post-operative | 57.4 ± 31.1 | 26.9 ± 25.3 | 37.6 ± 19.6 | 57.6 ± 20.8 | 77.0 ± 16.3 |
|  | p-value pre vs post | .433 | .913 | .570 | .022 | **.020** |
|  | Lean | 65.5 ±18.7 | 30.2 ± 17.9 | 37.7 ± 22.1 | 49.4 ± 17.2 | 66.3 ±15.1 |
|  | p-value lean vs pre-operative | .235 | .566 | .255 | .271 | .074 |
|  | p-value lean vs post-operative | .320 | .498 | .909 | .143 | .108 |
| ***Satiety*** | ***p-value between groups*** | .211 | .081 | **.003** | **.004** | .117 |
|  | Pre-operative | 29.9 ± 24.4 | 61.2 ± 25.2 | 56.8 ± 21.1 | 45.6 ± 22.6 | 35.9 ± 25.7 |
|  | Post-operative | 30.1 ± 26.7 | 63.3 ± 31.5 | 49.6 ± 21.2 | 37.8 ± 18.8 | 25.6 ± 22.9 |
|  | p-value pre vs post | .845 | .850 | .145 | .151 | .211 |
|  | Lean | 20.1 ± 18.1 | 53.5 ±19.8 | 40.8 ± 15.4 | 28.6 ± 14.4 | 22.8 ± 12.9 |
|  | p-value lean vs pre-operative | .099 | .130 | **.004** | **.004** | .014 |
|  | p-value lean vs post-operative | .190 | .208 | .071 | .395 | .517 |
| ***Prospective Food Consumption*** | ***p-value between groups*** | .688 | .094 | .205 | .360 | .173 |
|  | Pre-operative | 66.4 ± 21.8 | 43.0 ± 25.8 | 46.6 ± 23.7 | 52.7 ± 22.1 | 64.0 ± 21.2 |
|  | Post-operative | 68.5 ± 23.2 | 34.8 ± 28.6 | 50.2 ± 20.4 | 60.1 ± 19.1 | 70.6 ± 24.9 |
|  | p-value pre vs post | .570 | .320 | .306 | .170 | .187 |
|  | Lean | 70.2 ± 13.5 | 47.3 ± 20.3 | 54.8 ± 17.9 | 60.6 ± 17.7 | 70.3 ± 14.3 |
|  | p-value lean vs pre-operative | .369 | .512 | .112 | .091 | .155 |
|  | p-value lean vs post-operative | .732 | .124 | .405 | .854 | .964 |
| ***Desire to eat*** | ***p-value between groups*** | .113 | .751 | .679 | .111 | **.039** |
|  | Pre-operative | 61.6 ± 26.7 | 29.7 ± 24.2 | 35.8 ± 23.1 | 46.4 ± 25.1 | 58.7 ± 24.8 |
|  | Post-operative | 54.3 ± 32.7 | 33.2 ± 25.1 | 46.6 ± 22.3 | 61.9 ± 19.2 | 77.6 ± 17.9 |
|  | p-value pre vs post | .193 | .983 | .102 | .055 | **.035** |
|  | Lean | 68.5 ± 18.2 | 30.2 ± 18.1 | 41.8 ± 22.7 | 49.2 ± 20.9 | 67.6 ± 17.3 |
|  | p-value lean vs pre-operative | .253 | .815 | .233 | .579 | .117 |
|  | p-value lean vs post-operative | .110 | .709 | .527 | .038 | .050 |
